# Bad words prevail: Negatively charged Chinese characters accelerate attentional selection and preoccupy cognitive resources for consolidation

**DOI:** 10.3758/s13414-019-01758-9

**Published:** 2019-06-04

**Authors:** Shih-Yu Lo, Yuan-Sheng Wang

**Affiliations:** 1 Institute of Communication Studies, National Chiao Tung University, 1001 University Road, Hsinchu, 300 Taiwan; 2 Center for General Education, National Chiao Tung University, Hsinchu, Taiwan; 3 Degree Program of Science, College of Science, National Chiao Tung University, Hsinchu, Taiwan

**Keywords:** Visual perception, Attention: selective, Visual working memory

## Abstract

Previous studies have produced contradictory findings regarding whether emotion exerts facilitative effects or inhibitory effects on perception. In the present study, we hypothesized that attention can be separated into the initial selection stage and the latter consolidation stage, and emotion plays a different role in each of these two stages. To test this hypothesis, we adopted the dual-stream rapid serial visual presentation paradigm (Goodbourn & Holcombe, Journal of Experimental Psychology: Human Perception and Performance, 41(2), 364–384, 2015), which provided separate measurements for selection latency (how delayed the attentional selection process is) and efficacy (how much information can be successfully consolidated for conscious report). The results suggested emotion’s dual role on perception. Firstly, the presence of negatively charged visual targets (which were Chinese characters in the present study) accelerated attention selection, and the acceleration effect could spread to different locations in the visual field. Secondly, negatively charged characters preoccupied attentional resources for consolidation, yielding location-specific facilitative and inhibitory effects.

## Introduction

### Emotion and visual perception

A major function of our cognitive system is to create mental representations for external objects. In general, physically salient objects can gain processing priority (Itti & Koch, 2000). In addition, our inner states, including the task goal (Folk, Leber, & Egeth, 2008; Folk, Remington, & Johnston, 1992; Lo, 2018), knowledge (Biederman, Mezzanotte, & Rabinowitz, 1982; Oliva & Torralba, 2007; Palmer, 1975), and emotion, can also modulate this object-to-representation process.

The present study focuses on the interaction between emotion and the perceptual system. As Simon (1967) points out, the chief function of emotion is to reorder processing priorities. Specifically, stimuli that signal potential dangers will be prioritized. Empirical evidence for how emotion modulates perception has been shown in miscellaneous behavioral studies (Fox, Russo, Bowles, & Dutton, 2001; Öhman, Flykt, & Esteves, 2001; Tsuchiya, Moradi, Felsen, Yamazaki, & Adolphs, 2009; Van Damme, Crombez, & Notebaert, 2008, Yang, Zald, & Blake, 2007) and neuroimaging studies (Bradley et al., 2003; Junghofer et al., 2006; Junghofer, Schupp, Stark, & Vaitl, 2005; Sabatinelli, Bradley, Fitzsimmons, & Lang, 2005; Vuilleumier, 2005). For example, in the study by Phelps, Ling, and Carrasco (2006), the participants’ perceptual judgment for a peripherally presented Gabor patch was facilitated by a central fearful face that was presented prior to the target, evidenced by a contrast sensitivity increase of 22% at threshold. In this case, a fearful face signaled a potential danger, which might have elevated the observer’s arousal state, leading to a better performance. The facilitative effect of emotion on perception was further corroborated by event-related potential (ERP) studies (Pourtois, Grandjean, Sander, & Vuilleumier, 2004; Pourtois, Thut, Grave de Peralta, Michel, & Vuilleumier, 2005; Zinchenko, Kanske, Obermeier, Schroger, & Kotz, 2015). For example, Zinchenko et al. (2015) demonstrated that the facilitative effect of negative emotion on cognitive functions could be manifested in the ERP component that arose 100 ms after stimulus onset.

Paradoxically, emotion-charged stimuli sometimes lead to an inhibition effect. In a series of studies that investigated emotion-induced blindness (EIB) (Most, Chun, Widders, & Zald, 2005), observers’ detection performance regarding a target picture deteriorated when a task-irrelevant emotional distracter preceded the target picture (Kennedy, Rawding, Most, & Hoffman, 2014; Most, Chun, Johnson, & Kiehl, 2006; Most et al., 2005; Most & Jungé, 2008; Wang, Kennedy, & Most, 2012). In Most and Wang’s (2011) study, for example, the mean accuracy of the perceptual judgment task decreased by approximately 13% in the negative condition with respect to the neutral condition. So why does EIB occur? A review by McHugo, Olatunji, and Zald (2013) suggests that EIB might be a special case of the perceptual phenomenon known as the attentional blink (AB). The AB occurs when the observer has to identify two targets (T1 and T2) among a stream of rapid, serially presented visual stimuli, and the detection of the second target (T2) is disrupted when T2 is presented roughly 180–450 ms after T1 (Raymond, Shapiro, & Arnell, 1992). A possible explanation for the AB effect is that target identification requires attention, and attention is not available for T2 when it is presented too close to T1, while the attention-demanding mechanism is still engaged with T1 (Chun & Potter, 1995). Presumably, the AB effect occurs only when there are two targets. When there is only one target, the AB effect should not occur, but an emotion-charged distracter presented prior to the target might also attract attention and automatically become a “T1,” impairing the target identification performance and causing the EIB effect.

### Two-stage models for attention

As mentioned above, some studies demonstrated a facilitative effect of emotion on perception (e.g., Phelps et al., 2006), while others demonstrated opposite effects (e.g., Most et al., 2005). To reconcile this contradiction, Bocanegra and Zeelenberg (2009) manipulated the cue-target interval and showed a facilitative effect of an emotion cue on target identification with a long cue-target interval (1,000 ms), and an inhibitory effect with short intervals (50 ms and 500 ms). To explain the cue-target interval effect, Bocanegra and Zeelenberg (2009) speculated that short and long cue-target intervals probed two different stages of attentional processing, and emotional stimuli exert distinct effects on these two separate stages.

Researchers have postulated different versions of two-stage models (Bowman & Wyble, 2007; Chun & Potter, 1995; Goodbourn & Holcombe, 2015; Kanwisher, 1987) that account for the attentional operation on rapidly presented stimuli. These models generally involved a high-capacity sensory processing stage, followed by a capacity-limited stage that consolidates the sensory information into stabilized representations for conscious report. In the study of Bocanegra and Zeelenberg (2009), the authors speculated that the presence of emotional stimuli may facilitate the high-capacity sensory processing stage (stage 1), which could carry over to other stimuli that were presented within a long range of time, leading to facilitative effects for stimuli that are presented long after the emotional distracters. However, emotional stimuli also preoccupy resources for consolidation (stage 2), resulting in impairment for consolidation of the stimuli that are presented closely to the emotional distracters.

Although the conceptual framework of the two-stage model is commonly seen in previous studies, few studies have provided a method that can separately probe the two separate stages. In a recent study, Goodbourn and Holcombe (2015) developed an experimental paradigm that could separately manifest the parallel nature of the initial sensory stage, and the serial nature of the latter consolidation stage, which they termed the “parallel activation and serial tokenization” model of attention. In the critical conditions of this study, participants viewed two letter streams of rapid serial visual presentation (RSVP) and were instructed to monitor one (single-target condition) or both (dual-target condition) streams. Two flashing rings were presented in the middle of the trial, and the task was to identify the letters that co-occurred with the ring cues. Serial position errors (SPEs) were estimated by the temporal lag between the reported letters and the target letters. For example, if the participant accurately reported the target letter, then the SPE would be 0; if the subject reported the letter that was presented one item after the target letter, then the SPE would be +1. The results of the study revealed that the single-target condition and the dual-target condition yielded different SPE distributions. To investigate the SPE differences, the SPE distributions were then fitted with a mixture model that was composed of a Gaussian distribution and a uniform distribution. The Gaussian distribution was comprised of the SPEs derived from perceived targets, and the uniform distribution was comprised of the SPEs derived from random guessing responses. The rationale of this mixture model is that when participants viewed a series of letters, all the letters activated their *type* representations, which were fleeting and inaccessible for conscious report. The appearance of the ring cues triggered the “selection” process, and only the selected representations could be further consolidated in the visual short-term memory to become *token* representations for conscious report. The Gaussian distribution in the mixture model was comprised of the selected representations. A higher proportion of Gaussian distribution indicates more trials with successful selection and consolidation; thus, Goodbourn and Holcombe (2015) defined the proportion of the Gaussian distribution as the selection *efficacy*. The Gaussian distribution is determined by two parameters, the mean and the standard deviation, and they can be estimations of selection *latency* and *imprecision.*^1^

Compared to single-target selection, dual-target selection did not affect the selection time, as indicated by equivalent selection latency and selection imprecision between the single-target condition and the dual-target condition. Therefore, simultaneously presented stimuli activate their type representations in a parallel fashion, and the appearance of ring cues triggered the process that selected the two targets in a parallel fashion as well. The cost of processing two targets arises at the consolidation stage, evidenced by the lower selection efficacy in the dual-target condition than in the single-target condition. When participants had to consolidate the two selected target representations, they tended to make a reasonable response for one target and guess for the other. Which one could be prioritized? According to Goodbourn and Holcombe’s (2015) finding, the stimulus on the left side was prioritized because the target on the left side yielded a higher selection efficacy than the target on the right side.

### Goal of the present study

The effect of emotion on attention could be facilitative (e.g., Phelps et al., 2006) or inhibitory (e.g., Most & Wang, 2011). Bocanegra and Zeelenberg's (2009) study showed that the cue-target interval could be a possible cause for this controversy, but further research is needed to examine the underlying mechanism of the cue-target interval effect. In the present study, we examined the emotional effect on perception, based on the paradigm developed by Goodbourn and Holcombe (2015), which could separately probe the initial selection stage and the latter consolidation stage of attentional processing. More specifically, if stimulus emotion affects the initial selection stage by modifying the selection time, different latency (mean of selection times) or imprecision (standard deviation of selection times) values for stimuli with different emotions should be observed; if stimulus emotion affects the subsequent consolidation stage, the only index that could be affected by stimulus emotion should be the selection efficacy.

A challenge in this study was to develop a task where participants were able to identify briefly presented stimuli that are emotionally charged. We chose Chinese characters to achieve this goal. A Chinese character is considered a morpheme, which, in a language, is the smallest unit that possesses a meaning. Its morphemic feature enables it to possess a denotation, which is the literal meaning of the character, and a connotation, which implies a particular emotional value.

This study is made up of three experiments. Experiment 1 was a replication of Goodbourn and Holcombe’s (2015) study, so English letters were used as stimuli. The critical experiments were Experiments 2 and 3, where Chinese characters with neutral or negative connotations were used.

## Experiment 1

### Methods

#### Participants

The experimental protocols in this study were approved by the Research Ethics Committee for Human Subject Protection of National Chiao Tung University. Eight people (three males) in Experiment 1 (age range: 20–21 years, median = 20 years) with normal or corrected-to-normal vision participated in this experiment, and all gave their written consent before participating. The data from one participant (female, 20 years of age) were excluded because the mixture-modeling analysis (details will be described below) suggested that she made random reports on 99% of the trials. Subsequent statistical analyses were based on the other seven participants (three males; age range: 20–21 years; age median = 20 years).

The aim of Experiment 1 was to replicate Goodbourn and Holcombe’s (2015) study. The main finding in their study was the higher efficacy value for the left target than the right target. To achieve the effect size of the left-side advantage observed in their bilateral condition in Experiment 2, where stimuli were bilaterally presented and the effect size *d* was 1.81, a minimum sample size of six was required to achieve 90% power, according to power analysis performed by G*Power 3 (Faul, Erdfelde, Lang, & Buchner, 2007).^2^ Furthermore, in Goodbourn and Holcombe’s original (2015) study, they initially recruited six participants for each experiment and then replicated the results of these six participants by adding another group of 20 observers for each experiment, in the same study. Therefore, a sample size of seven should be sufficient to manifest the left-side benefit on efficacy with English letters.

#### Apparatus

All three experiments in this study were programed in MATLAB r2014b (32-bit) with Psychtoolbox-3 extensions (Brainard, 1997; Pelli, 1997). Visual stimuli were displayed on a 17-in. CRT monitor (Mitsubishi i-TECH IF700 CRT Monitor) with a spatial resolution of 1,024 × 768 pixels and a refresh rate of 85 Hz. A viewing distance of 66 cm was maintained with a chinrest. An Arrington MHU 03 eye tracker was attached to the chinrest. However, due to a technical error, the sample rate of eye-gaze location was not high enough to differentiate fixations and saccades. In all experiments in this study, the participants were informed of an eye-tracking device that recorded their eye movements. This encouraged participants to concentrate, but no data from fixations and saccades were available for analysis.

#### Stimuli and procedure

This was a replication of an experiment in Goodbourn and Holcombe’s (2015) study. Two streams of letter RSVP were presented either on the upper two quadrants or the lower two quadrants of the display, with the letter center having an eccentricity of 4°. The letters were white (95 cd/m^2^) uppercase English letters rendered in Menlo font. The height of the letter was approximately 4° and the width was 2°.

**Fig. 1:**
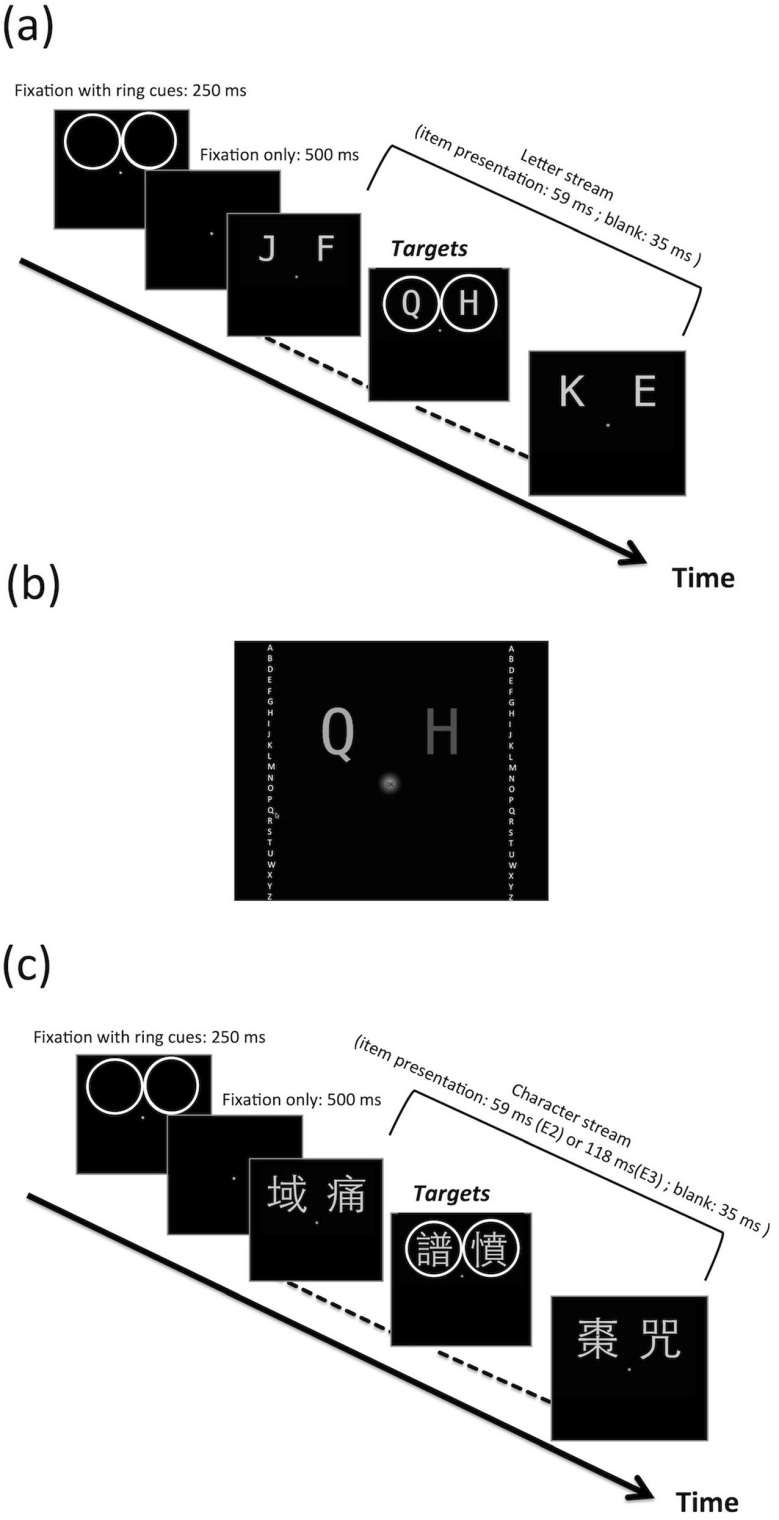
Schematic representations of the (**a**) rapid serial visual presentation (RSVP) streams, (**b**) response display in Experiment 1, and (**c**) RSVP streams in Experiments 2 and 3. Each trial started with a 250-ms fixation-with-ring-cues display, and then a 500-ms fixation-only display. The letter or character stream consisted of 24 stimulus frames of 59 ms (Experiments 1 and 2) or 118 ms (Experiment 3) separated by a 35-ms blank interval. The targets were one of the middle 12 frames (the seventh to the 18th inclusive) and designated by rings. The sizes and brightness levels of the contours in these diagrams are for illustration purposes, and they are not exactly identical to the contours used in the experiments. Please see the main text for the exact stimulus parameters used in the experiments

Figure 1a provides an illustration of the experimental stimuli. Each stream began with a 250-ms fixation-with-ring-cues display, where a central fixation dot (55 cd/m^2^) with a diameter of 0.25° was presented with one or two rings (40 cd/m^2^) with a diameter of 5.5° in the upper two quadrants or lower two quadrants. The function of the rings was to mark whether this trial was a one-target trial (one ring) or a two-target trial (two rings). After a fixation-only display of 500 ms, two letter streams appeared on the upper or the lower two quadrants, centered at an eccentricity of 4°. For each stream, 24 letters (C and V were avoided due to confusion with other letters) were presented, with the order randomized without replacement. Each letter was presented for 59 ms, separated by a 35-ms blank. In the *one-target condition*, a ring cue with a diameter of 5.5° circled around the target letter. The possible target letter appeared between the seventh and the 18th item (inclusive) of the stream. In the *two-target condition*, two ring cues were presented simultaneously circling around the two target letters in the two streams.

After the letter stream RSVP was the response display (Fig. 1b). On the response display in the one-target condition, a vertical column comprised of white letters A–Z except for C and V (55 cd/m^2^) was presented on the corresponding target side, with a distance of 9° with respect to the central vertical meridian. Each letter was 0.4° in width and 0.7° in height. At the location where the target stream was presented, there was an underscore, informing the participant of the location he or she was about to respond to. Once the participant clicked on any letter on the letter column, the underscore would change to the letter the participant had just clicked on. Then, the participant had to click on the OK button at the center to confirm the answer. The OK button was a black word “OK” superimposed with a Gaussian blob that peaked at a luminance value of 55 cd/m^2^, with a sigma value of 0.3°. In the two-target condition, the participant’s response order for the two targets was randomized (left side first on some trials and right side first on others). The letter column and the underscore for the first side (e.g., the left side in the trials where the participant had to make the response to the left target first) was always white (55 cd/m^2^), and those for the second side were gray (8 cd/m^2^). After completing the response for the first side, the letter column and the underscore on the first side would turn gray, and those on the second side would turn white.

The participant first completed a 20-trial practice session. More practice trials were possible if the participant was not confident enough to begin the experimental session. The experimental session consisted of 128 trials, with 64 of these in the *one-target condition* and the other 64 in the *two-target condition*. In the one-target condition, the target stream was on the left side on half of the trials and the right side on the other half. The order of the trials in different conditions was randomized. The participants completed the experiment within approximately 1 h.

### Analysis

#### Serial position error

The serial position error (SPE) was defined by the temporal position difference between the target letter and the reported letter. For example, if the participant accurately reported the target letter, the SPE would be 0; if the participant reported the letter that was presented one item after the target, the SPE would be +1; if the participant reported the letter that was presented one item prior to the target, the SPE would be −1. For each condition, a distribution of SPEs could be collected.

#### Mixture model

For any given trial, the participant might have reported a letter he or she really perceived, which was referred to as an effective trial. Alternatively, the participant might have guessed and made a random response, which was referred to as a guessing trial. Presumably, SPEs for effective trials should distribute according to a Gaussian distribution, and SPEs for guessing trials should distribute uniformly. Based on these assumptions, we fitted the data with a mixture model comprising a Gaussian distribution and a uniform distribution, formulated as follows:

1 $$ f\left(x,{p}_T,\mu, \sigma \right)=W(x)\left[\frac{p_T}{C_N}N\left(x,\mu, \sigma \right)+\frac{\left(1-{p}_T\right)}{C_U}U(x)\right]. $$

In this formula, *x* is a given SPE value*, f* indicates the probability of this given SPE *x*, *p*_T_ is the index of *selection efficacy,* represented by the proportion of the Gaussian distribution *N* (*x*,μ,σ). The mean and the standard deviation of the Gaussian distribution were μ and σ, which were respectively the indices of *selection latency* and *selection imprecision*. The term *U*(*x*) is the uniform distribution of guessing trials.

Selection *latency* and *imprecision* served as indices of how fast and how reliable the selection process took place. If selection occurred very soon after the ring cues appeared, the latency value should be small; otherwise it should be large. If the selection time varied a lot from trial to trial, the imprecision value should be large; otherwise it should be small. Efficacy served as an index of the amount of trials where the consolidation process was complete. For trials where the consolidation process was incomplete, no consolidated representation was available in the short-term memory, leading to a guessing response. A high proportion of guessing responses would lead to a low efficacy value.

In Equation 1, there was a window function *W*(*x*). This term was necessary due to the design of the present experiments, where possible SPE values ranged from −17 to 17. The function of *W*(*x*) is defined as:

2 $$ W(x)=\Big\{{\displaystyle \begin{array}{ll}0& x\le S{P}_l\\ {}S{P}_l+x& -S{P}_l<x<\left(1-S{P}_f\right)\\ {}S{P}_l-S{P}_f+1& \left(1-S{P}_f\right)\le x\le \left(S{P}_t-S{P}_l-1\right)\\ {}S{P}_t-S{P}_f-x& \left(S{P}_t-S{P}_l-1\right)<x<\left(S{P}_t-S{P}_f+1\right)\\ {}0& x\ge \left(S{P}_t-S{P}_f+1\right)\end{array}} $$

In the equations above, SP_*f*_ and SP_*l*_ refer respectively to the first and the last serial positions where the target could appear, and SP_*t*_ is the total number of items in the stream. The three values were 7, 18, and 24, respectively, in the current study.

The window function (Eq. 2) can be understood in the following way: To get an SPE of 1, the serial position of the target and that of the reported letter could be, respectively, 7 and 8, 8 and 9, etc. To get an SPE of 17, the serial positions of the target and that of the reported letter could only be 7 and 24, where the serial position of 7 is the first possible target location, and 24 is the last position the observer could possibly report. In general, extreme SPEs can only occur on few trials, leading to an uneven distribution for the window function *W(x)*.

As the integral of the overall mixture function must be equal to 1, normalizing constants must be applied to Eq. 1: *C*_*N*_ is the normalizing constant for the windowed Gaussian distribution:

3 $$ {C}_N=\sum \limits_{x=-S{P}_l}^{S{P}_t-S{P}_f+1}W(x)N\left(x,\mu, \sigma \right) $$

and *C*_*U*_ is the normalizing constant for the windowed uniform distribution:

4 $$ {C}_U=\sum \limits_{x=-S{P}_l}^{S{P}_t-S{P}_f+1}W(x)U(x) $$

#### Model fitting

**Fig. 2:**
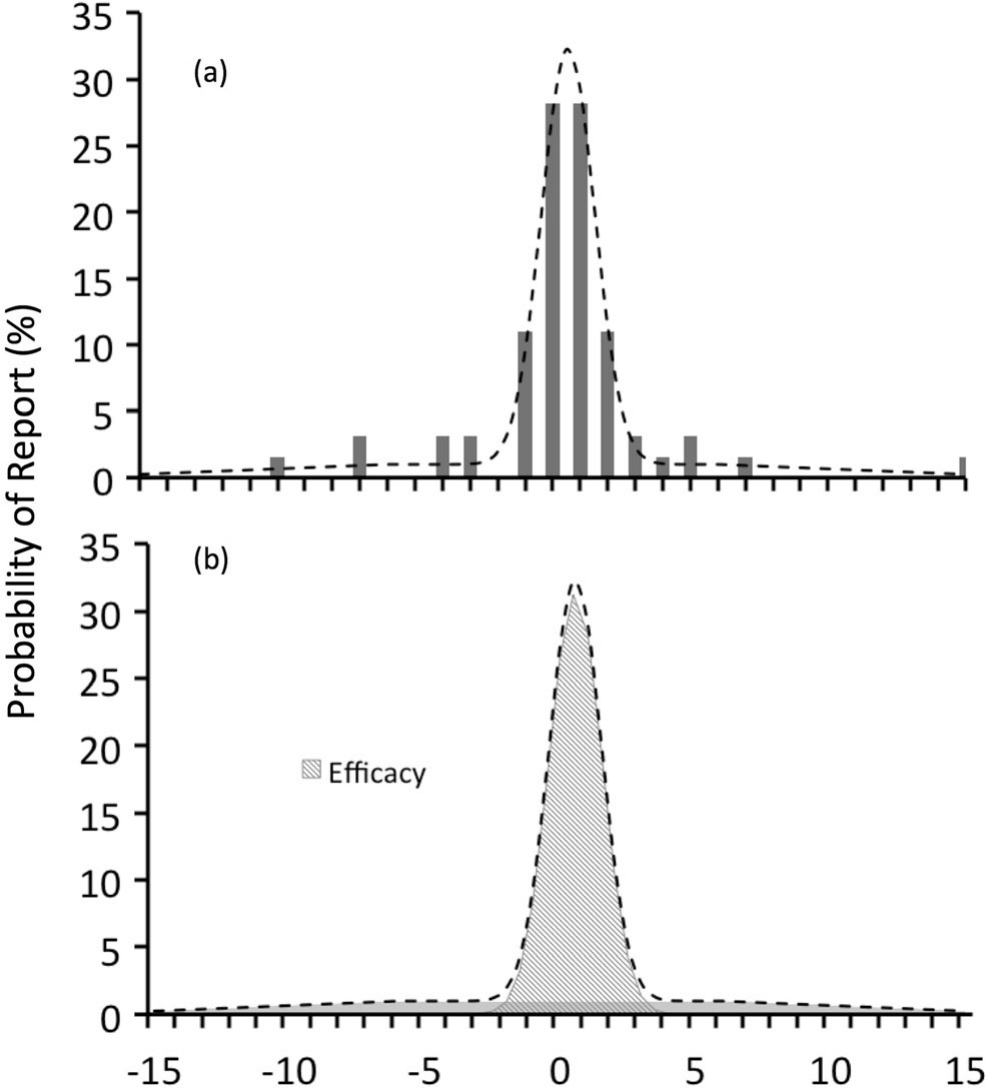
Example histogram of serial position errors and model fit. (**a**) The histogram shows an example distribution (the responses for the right stream of the two-target condition, from participant AKC), with the dashed line representing the best-fitting mixture model, of which the components are shown in panel b. (**b**) The two components are as follows: the Gaussian component (the bell-shaped gray surface), which represents the proportion of effective trials; the random-report component (the flat-shaped gray surface), which represents the proportion of guessing trials

For each condition, we fitted the SPE data with three free parameters (*p*_*T*_, *μ*, *σ*) in the mixture model (Eq. 1). A maximum likelihood method implemented by the *mle* comment in Matlab was used to estimate the parameters. The three parameters were estimated 30 times with different starting values. Figure 2 shows an exemplar SPE distribution of Experiment 1 (SPEs in the right stream in the two-target condition from participant AKC), with the raw data in Fig. 2a, and the fitted model in Fig. 2b.

### Results and discussion

We replicated the results of Goodbourn and Holcombe’s (2015) study. The selection latency and imprecision did not significantly differ between the one-target condition and two-target conditions. For selection efficacy, there was an interaction, whereby the efficacy value for the left side was significantly higher than the right side in the two-target condition but not in the one-target condition.

**Fig. 3:**
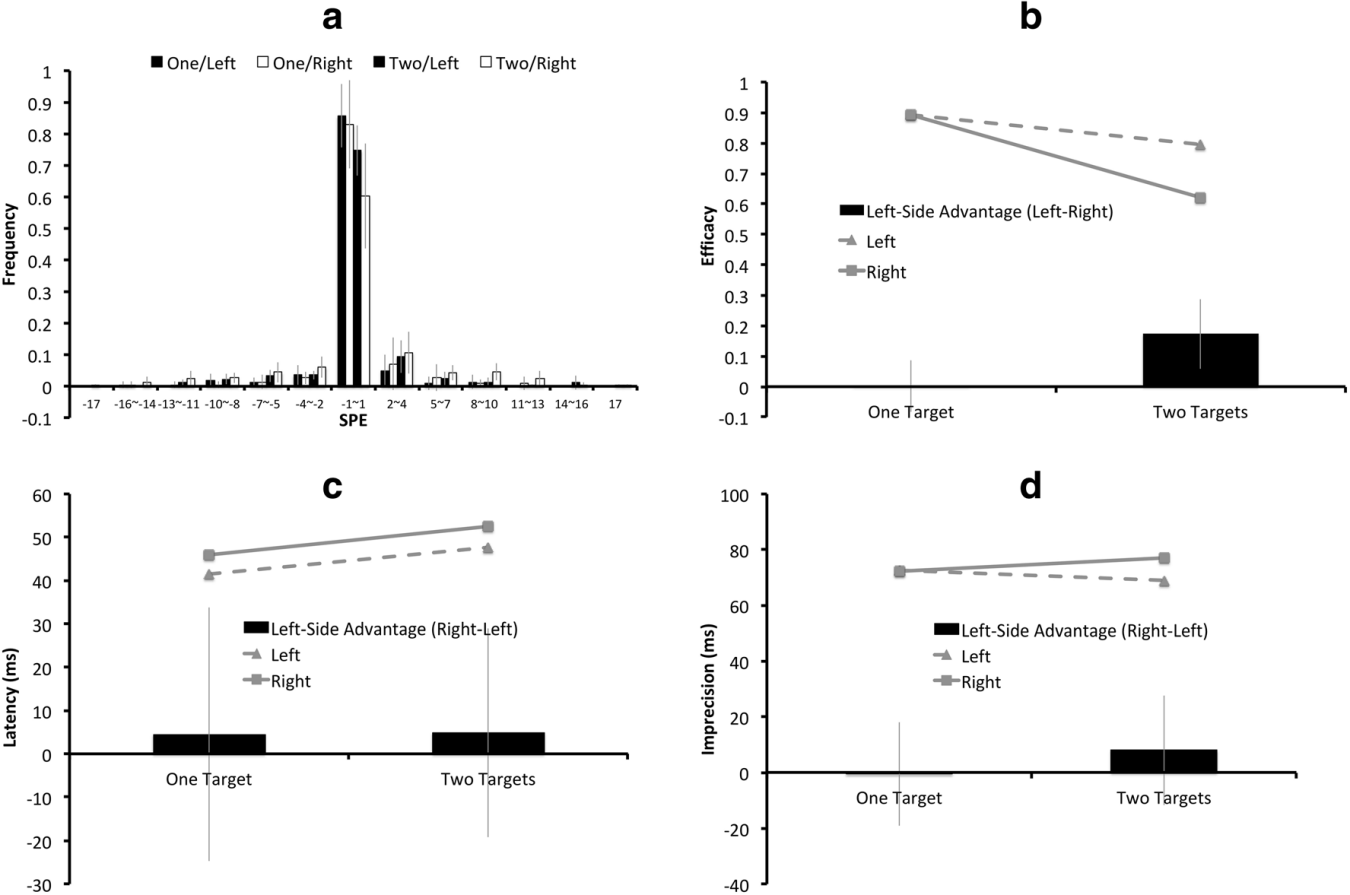
Results of Experiment 1: (**a**) The serial position error (SPE) distributions for the one-target/left, the one-target/right, the two-target/left, and the two-target/right conditions are plotted from left to right for each SPE bin. The (**b**) mean efficacy, (**c**) latency, and (**d**) imprecision values for the left targets are represented by triangular markers connected by dashed lines, and those for the right targets are represented by square markers connected by solid lines. In Figs. (**b**)**–**(**d**), the black columns indicate the “left-side advantages,” derived from the differences on the (**b**) efficacy, (**c**) latency, or (**d**) imprecision value between the left-target condition and the right-target condition, with a positive value indicating an effect favoring the left side. The error bars indicate 95% CIs of the left-side advantages

The SPEs for each condition are shown in Fig. 3a, with the error bars indicating the 95% confidence intervals (CIs) across the seven participants. Figure 3b–d illustrate the mean efficacy, latency, and imprecision values for the left targets and the right targets, separately in the one-target condition and the two-target condition. The black columns in Fig. 3b–d represent the “left-side advantages” derived from the differences between the left-target condition and the right-target condition. A positive value indicates an effect favoring the left target, which could be a higher efficacy value, or a lower latency/imprecision value for the left target with respect to the right target. The error bars indicate the 95% confidence intervals (CIs) of the “left-side advantages” across the seven participants. The statistical analyses on efficacy, latency, and imprecision values are described below.

#### Efficacy

The efficacy values were subjected to a repeated-measures ANOVA, with two within-subject factors: target number (one target or two targets) and target side (left or right). There were significant effects of target number (*F*(1, 6) = 19.59, *p* = .004, *η*_*partial*_^*2*^ = .77) and target side (*F*(1, 6) = 7.36, *p* = .03, *η*_*partial*_^*2*^ = .55), as well as a significant interaction between the two factors (*F*(1, 6) = 11.22, *p* = .02, *η*_*partial*_^*2*^ = .65). In the one-target condition, there was no significant difference (*t*(6) = 0.02, *p* = .98, *Cohen’s d* = 0.008) between the efficacy value for the left target (*M* = .89) and for the right target (*M* = .89). In the two-target condition, the efficacy value for the left target (*M* = .80) was significantly higher (*t*(6) = 3.70, *p* = .01, *Cohen’s d* = 1.40) than that for the right target (*M* = .62).

#### Latency and imprecision

There was no statistically significant effect on latency or imprecision of target number, target side, or interaction between the two (all *ps*>.05, all *η*_*partial*_^*2’*^*s* < .15).

## Experiment 2

Experiments 2 and 3 were critical experiments of this study. Chinese characters with negative or neutral connotations were used to test how efficacy, latency, and imprecision varied with stimulus emotion.

### Methods

#### Participants

Twenty-five people (14 males) participated in Experiment 2 (age range: 20–38 years, median = 22 years).

The main research interest in this experiment was the effect of emotion (negative vs. neutral). We used the data in the study of Most and Wang (2011), where the participants were presented with two streams of RSVP as in the case of the present study, as the reference of our calculation of the required sample size. According to the power analysis performed with G*Power 3 (Faul, et al., 2007), a minimum sample size of 16 was required to achieve 90% power.^3^ We nevertheless decided to add more participants to provide more than sufficient power.

#### Procedure

Twenty-four Chinese characters with negative or neutral connotations were presented in the RSVP streams (Fig. 1c). The connotations for the two character streams on the left and the right sides could be neutral versus neutral, neutral versus negative, negative versus neutral, or negative versus negative. In the one-target condition, the participants had to monitor and report one of the two streams, which could be neutral or negative; in the two-target condition, the participants had to monitor and report both streams. For each stream in each trial, the 24 Chinese characters were randomly chosen from a pool of 120 Chinese neutral characters or a pool of 120 Chinese negative characters (see Appendix). The mean character frequency and the mean stroke count for the characters in the neutral pool and the negative pool were not significantly different according to the database published by National Language Committee, Ministry of Education of Taiwan (1998). The neutral and the negative pools were identical across different trials and different participants, but a different set of 24 characters was randomly chosen again for each stream in each trial. After completing the experiment, each participant was required to fill out a questionnaire with all the characters from the two pools, to confirm whether the characters in the pools were perceived to be negative or neutral. Using a 7-point Likert scale, participants were required to rate how positive a given character was.

Similar to Experiment 1, the response display consisted of one or two character columns, underscores, and the central OK button (Fig. 1b), but Chinese characters instead of English letters were presented. The 24 characters shown in the character column were the characters the participant had just viewed in the RSVP stream, but with a different order.

The participant first completed a 20-trial practice session. More practice trials were possible if the participant was not confident enough to begin the experimental session. The experimental session consisted of 512 trials, with 256 of these in the one-target condition and the other 256 in the two-target condition. In the one-target condition, the target stream was on the left side on half of the trials and the right side on the other half. For each side, the target connotations were neutral on half of the trials and negative on the other half. In the two-target condition, the emotional connotations for the left and the right target streams could be neutral versus neutral, neutral versus negative, negative versus neutral, or negative versus negative, with an equal number of trials for each combination. The order of the trials was randomized, and the participant completed the experiment on four separate sessions with 128 trials in approximately 1 h on each session. Participants were encouraged to take a short break every 32 trials.

All other experimental parameters were identical to those in Experiment 1.

### Results

The data analysis in this experiment was split into two steps. In step 1, the data of the one-target condition and the two-target condition were both included. In step 2, only the data in the two-target condition were included.

#### Overall analysis

**Fig. 4:**
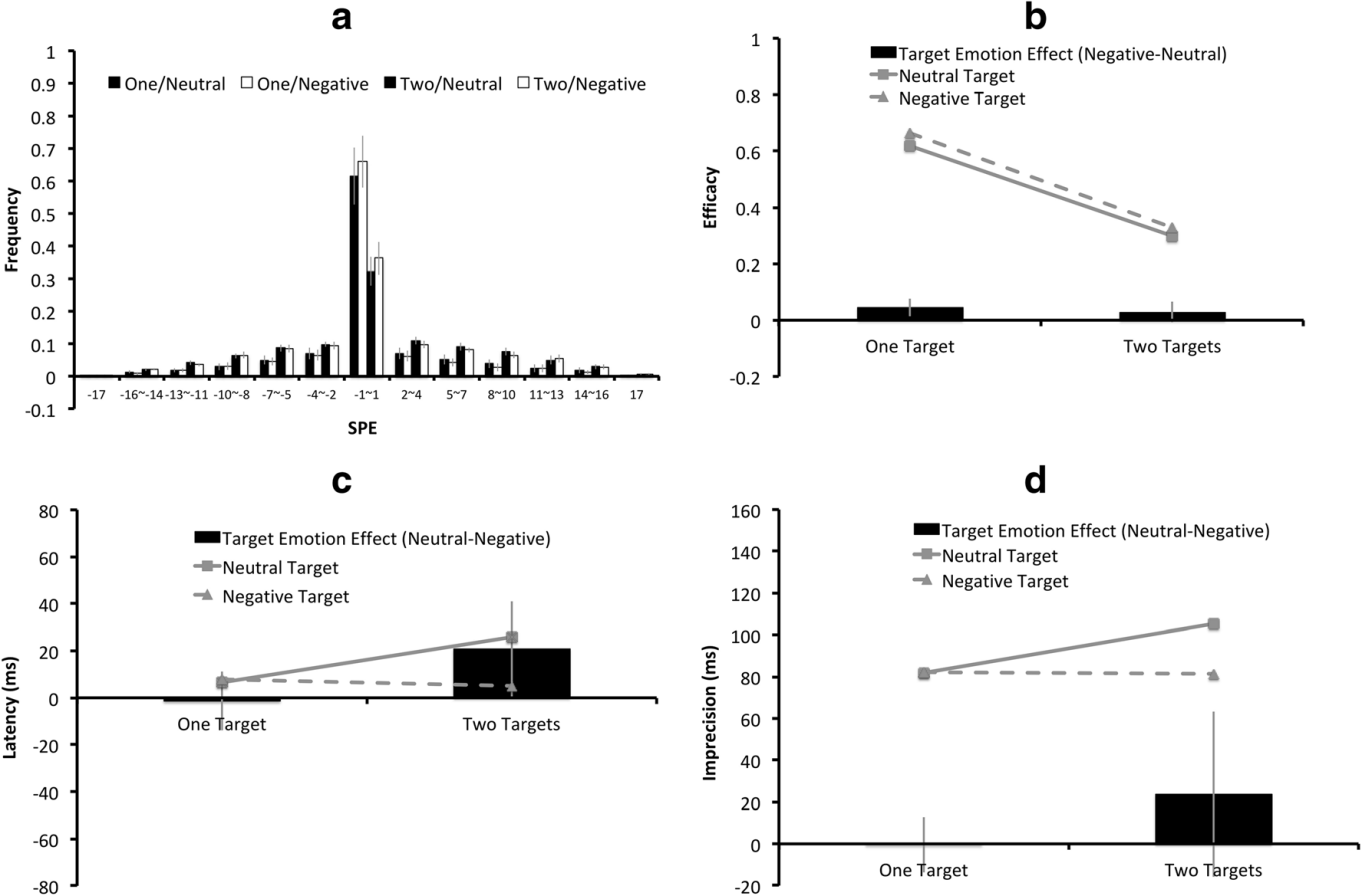
Results of the overall analyses of Experiment 2: (**a**) The serial position error (SPE) distributions for the one-target/ neutral, the one-target/negative, the two-target/neutral, and the two-target/negative conditions are plotted from left to right for each SPE bin. The (**b**) mean efficacy, (**c**) latency, and (**d**) imprecision values for the negatively charged targets are represented by triangular markers connected by dashed lines, and those for the neutral targets are represented by square markers connected by solid lines. In Figs. (**b**)**–**(**d**), the black columns indicate the “target emotion effects”, derived from the differences on the (**b**) efficacy, (**c**) latency, or (**d**) imprecision values between the negative-target condition and the neutral-target condition, with a positive value indicating an effect favoring the negative-target condition. The error bars indicate 95% CIs of the target emotion effects

The two critical factors of the present experiment were target number (one/two) and target emotion (neutral/negative), and thus the SPEs for the one-target/neutral, the one-target/negative, the two-target/neutral, and the two-target/negative condition were separately plotted in Fig. 4a, with the error bars indicating the 95% CIs across the 25 participants. Figure 4b–d illustrate the means for the aforementioned four conditions on efficacy, latency, and imprecision. In Fig. 4b–d, the black columns represent the “target emotion effects,” derived from the differences between the neutral-target condition and the negative-target condition. A positive value indicates an effect favoring the negatively charged target, which could be a higher efficacy value, or a lower latency/imprecision value for the negatively charged target with respect to the neutral target.

Unlike Experiment 1, target side did not yield any statistically significant effect on efficacy, latency, or imprecision. As the main interest of the present study was the “target emotion effect” instead of the “left-side advantage,” the data for the left target and the right target were collapsed in all the figures in this experiment for simplicity purposes. Nonetheless, the CIs of the left-side advantages will be provided separately for the one-target condition and the two-target condition.

The efficacy, latency, and imprecision values were subjected to a repeated-measures ANOVA, with three within-subject factors: target number (one or two), target emotion (neutral or negative), and target side (left or right). The statistical analyses on efficacy, latency, and imprecision values are described below.

- *Efficacy*. There was a significant effect of target number (*F*(1, 24) = 124, *p*< .001, *η*_*partial*_^*2*^ = .84), caused by higher efficacy value in the one-target condition (*M* = .64) than the two-target condition (*M* = .31). There was also a significant effect of target emotion (*F*(1, 24) = 11.03, *p* = .003, *η*_*partial*_^*2*^ = .31), where negatively charged characters yielded higher efficacy (*M* = .50) than neutral characters (*M* = .46). The 95% CIs of the left-side efficacy advantage of the one-target condition and the two-target condition were, respectively, [-0.1, 0.005] and [-0.04, 0.11], where a positive value represents an effect favoring the left side. Statistically, the effect of target side, and all the possible interactions among target side, target number, and target emotion, did not reach significance (all *ps*>.05, all *η*_*partial*_^*2’*^*s* < .15).
- *Latency and imprecision*. For both latency and imprecision, the main effects of target number, target emotion, target side, and their possible interactions did not reach statistical significance (all *ps*>.05, all *η*_*partial*_^*2’*^*s* < .15). The 95% CIs of the left-side latency advantage of the one-target condition and the two-target condition were, respectively, [-24 ms, 37 ms] and [-16 ms, 44 ms]; the 95% CIs of the left-side imprecision advantage of the one-target condition and the two-target condition were, respectively, [-19 ms, 59 ms] and [-6 ms, 70 ms], where a positive value represents an effect favoring the left side.

#### Two-target condition only

In the two-target condition, the connotations of the two targets on the two sides could be neutral versus neutral, neutral versus negative, negative versus neutral, or negative versus negative. Therefore, for any particular target, its own connotation was independent of the connotation of the other target on the other side of the display. In the following analysis, the emotional implication (connotation) of the target is referred to as the *target emotion*, and the emotional implication of the other target on the other side is referred to as the *neighbor emotion*. For example, in trials with a neutral stream on the left side and a negative stream on the right side, the target emotion for the left stream was neutral, and its neighbor emotion was negative.

**Fig. 5:**
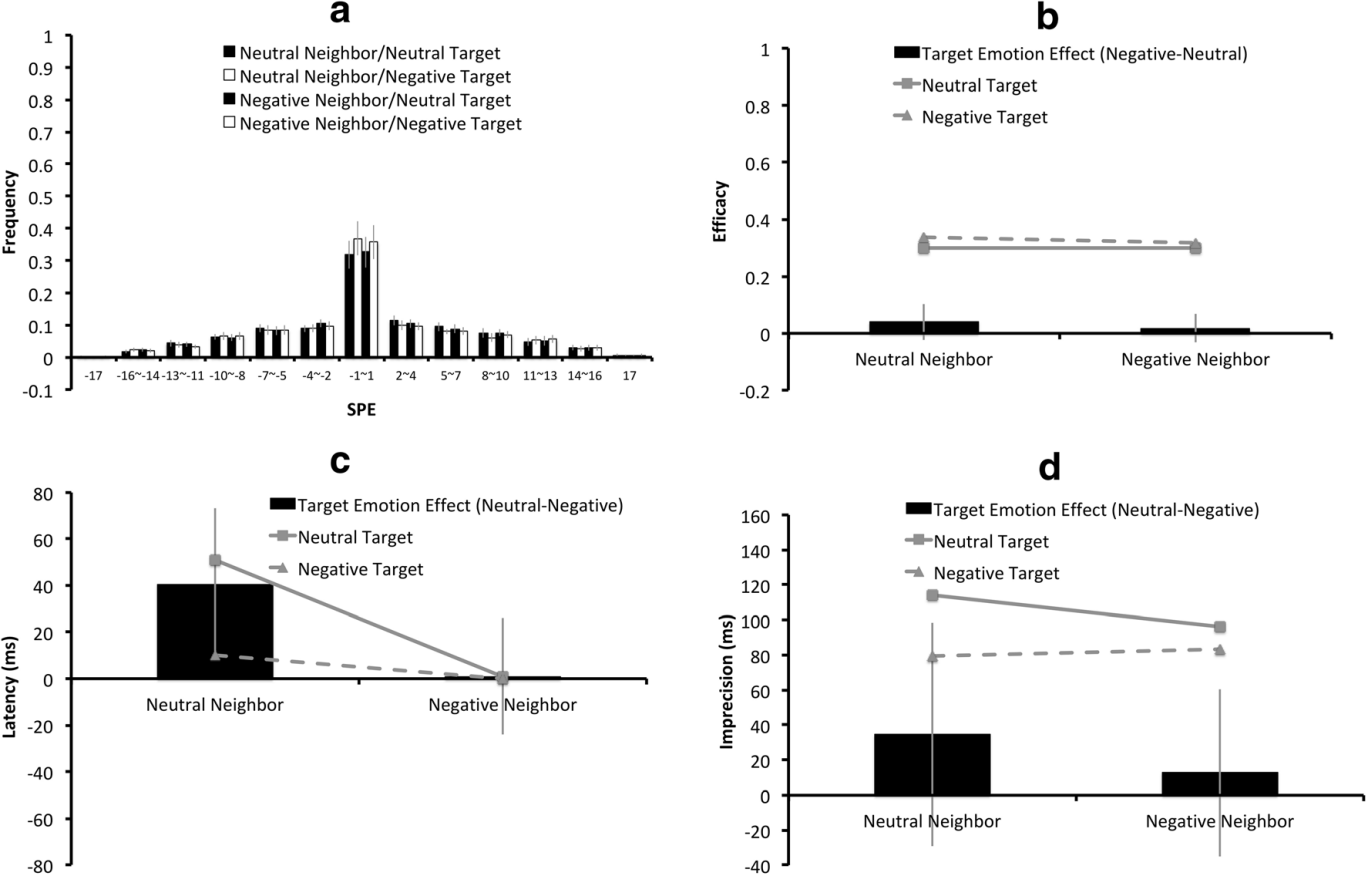
Results of the two-target condition only in Experiment 2: (**a**) The serial position error (SPE) distributions for the neutral-neighbor/neutral-target, neutral-neighbor/negative-target, negative-neighbor/neutral-target, and the negative-neighbor /negative-target conditions are plotted from left to right for each SPE bin. The (**b**) mean efficacy, (**c**) latency, and (**d**) imprecision values for the negatively charged targets are represented by triangular markers connected by dashed lines, and those for the neutral targets are represented by square markers connected by solid lines. In Figs. (**b**)**–**(**d**), the black columns indicate the “target emotion effects,” derived from the differences on the (**b**) efficacy, (**c**) latency, or (**d**) imprecision values between the negative-target condition and the neutral-target condition, with a positive value indicating an effect favoring the negative-target condition. The error bars indicate 95% CIs of the target emotion effects

The SPE distributions for the neutral-neighbor/neutral-target, the neutral-neighbor/negative-target, the negative-neighbor/neutral-target, and the negative-neighbor/negative–target conditions are plotted separately in Fig. 5a, with the error bars indicating the 95% CIs across the 25 participants. Figure 5b–d illustrate the means for each neighbor-target emotion combination on efficacy, latency, and imprecision. The black columns in Fig. 5b–d represent the “target emotion effects,” derived from the differences between the neutral-target condition and the negative-target condition.

The efficacy, latency, and imprecision values were subjected to a repeated-measures ANOVA, with three within-subject factors: target emotion (neutral or negative), target side (left or right), and neighbor emotion (neutral or negative).

- *Efficacy*. The effect of target emotion, neighbor emotion, target side, and any of their interactions did not reach statistical significance (all *ps*>.05, all *η*_*partial*_^*2’*^*s* < .15).
- *Latency*. There were significant effects of target emotion (*F*(1, 24) = 4.41, *p* = .046, *η*_*partial*_^*2*^ = .16) and neighbor emotion (*F*(1, 24) = 8.09, *p* = .009, *η*_*partial*_^*2*^ = .25). For neutral targets, the latency values for having a neutral neighbor and having a negative neighbor were 51 ms and 1 ms, respectively; for negatively charged targets, the latency values for the neutral neighbor and negative neighbor were 10 ms and 0 ms, respectively. Either being a negative target or having a negative neighbor, led to a lower latency value than its neutral counterpart. There was no significant main effect of target side, or significant interaction among target emotion, neighbor emotion, and target side ( *ps*>.05, all *η*_*partial*_^*2’*^*s* < .15).
- *Imprecision*. The effects of target emotion, neighbor emotion, target side, and any of their interactions did not reach statistical significance (all *ps*>.05, all *η*_*partial*_^*2’*^*s* < .15).

#### Rating results

For the 120 neutral characters and 120 negatively charged characters used in this experiment, we treated each character as a “subject,” and the mean rating value on a 7-point Likert scale derived from the 25 participants as the index of emotional positivity. A between-subject t-test showed that the negatively charged characters were indeed perceived to be more negative (*M* = 2.73) than the neutral characters (*M* = 4.22) (*t(238)* = 26.77, *p* < .0001, *Cohen’s d* = 3.46).

### Discussion

In this experiment, attentional selection process was shown to be accelerated as long as a negative target was present in the display, as evidenced by a shortened latency caused by a negative target or a negative neighbor. However, the effect of efficacy was equivocal. In the “overall” analysis, where the one-target condition and two-target condition were combined, negatively charged targets gain processing priority. In the two-target-only analysis, there was no significant effect of target emotion on efficacy. Therefore, the negative superiority effect mainly came from the one-target condition but not the two-target condition.

One discrepancy between Experiment 2 and Experiment 1 was the significant left-side advantage on efficacy in Experiment 1 but not Experiment 2. The two experiments were different in two aspects. First, English letters were used in Experiment 1 but Chinese characters were used in Experiment 2; second, emotion was manipulated in Experiment 2 but not Experiment 1. More specifically, Experiment 1 included only neutral items but Experiment 2 included neutral and negatively charged items. To conduct a fair cross-experimental analysis, all the conditions in Experiment 1 and only the one-target/neutral and the two-target/neutral-target/neutral-neighbor conditions in Experiment 2 were included, and “Experiment” was used as a between-subject factor with “target number” and “target side” as within-subject factors. The mixed-design ANOVA did show a significant interaction between Experiment and target side (*F*(1, 29) = 4.62, *p* = .04, *η*_*partial*_^*2*^ = .14 ). Therefore, for the discussions regarding the target side effect, Chinese characters and English letters are discussed separately in the rest of the paper.

## Experiment 3

In Experiment 2, although the effects of target emotion and neighbor emotion were significantly manifested on latency, the effect of target emotion on efficacy was only significant in the one-target condition but not the two-target condition. By inspecting the efficacy values (Fig. 5b), one can find that they were as low as approximately 0.3 in the two-target condition. Possibly, consolidating two targets that were presented for such a brief time was far too difficult. As consolidation occurred after selection, the very brief stimulus presentation time used in Experiment 2 might have been long enough for different stimulus emotions to exert differential effects on the initial selection stage, but caused a floor effect for the latter consolidation stage. In Experiment 3, a longer target presentation time would be used to test this possibility.

### Methods

#### Participants

Twenty-five people (13 males) participated in Experiment 3 (age range: 20–45 years, median = 22 years).

#### Procedure

The experimental parameters were identical to those in Experiment 2 except for the character presentation time. Instead of 59 ms, each character was presented for 118 ms.

### Results

#### Overall analysis

The efficacy, latency, and imprecision values were subjected to a repeated-measures ANOVA, with three within-subject factors: target number (one or two), target emotion (neutral or negative), and target side (left or right). As target side did not yield any significant effect on efficacy, latency, or imprecision, and the “left-side advantage” was not the main research interest of the present study, the data for the left side and the right side are collapsed in all the figures in this experiment for simplicity purposes. Nonetheless, The 95% CIs of the left-side advantages for the one-target condition and the two-target condition are reported, where a positive value indicates an effect favoring the left side.

**Fig. 6:**
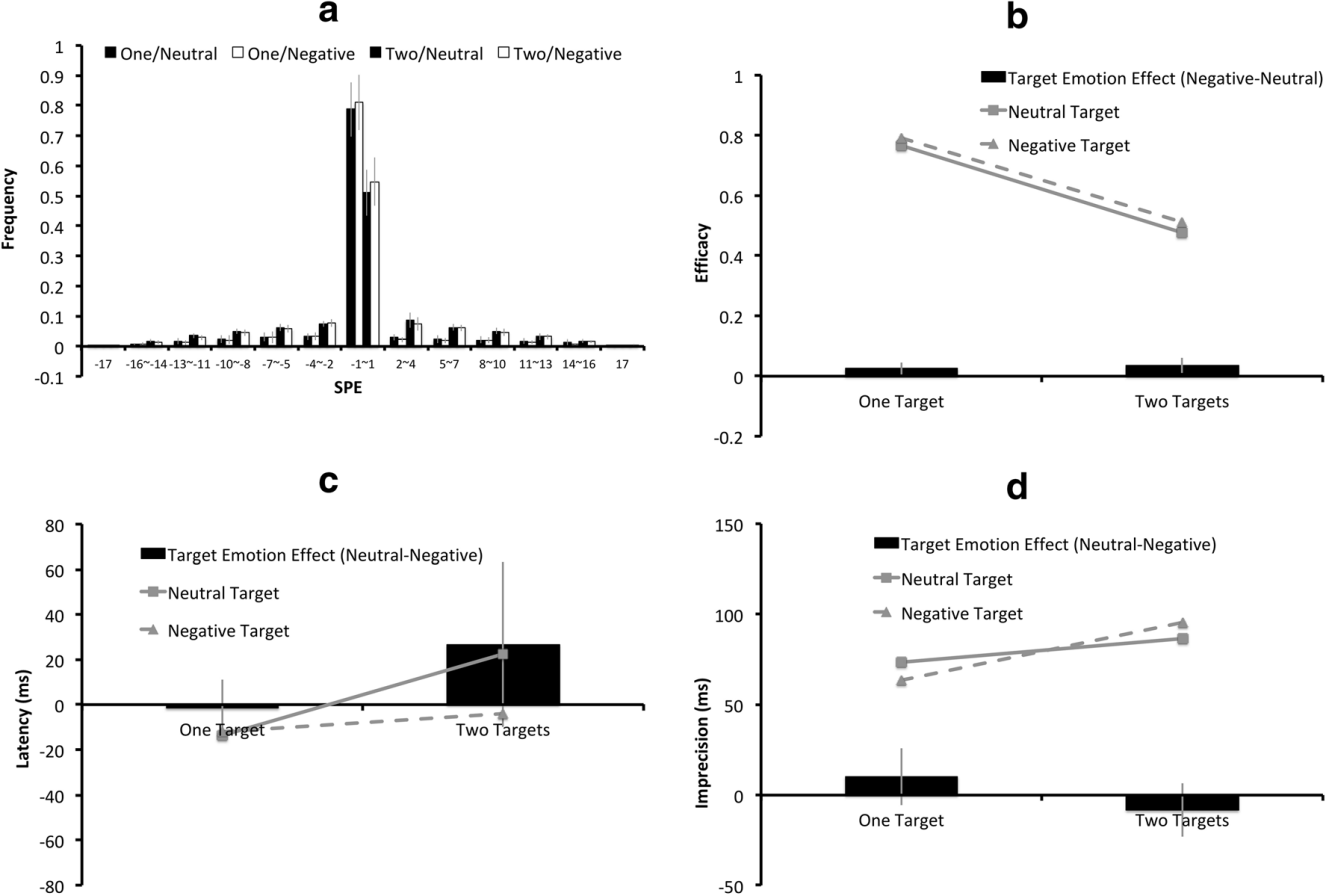
Results of the overall analyses of Experiment 3: (**a**) The serial position error (SPE) distributions for the one-target/neutral, the one-target/negative, the two-target/neutral, and the two-target/negative conditions are plotted from left to right for each SPE bin. The (**b**) mean efficacy, (**c**) latency, and (**d**) imprecision values for the negatively charged targets are represented by triangular markers connected by dashed lines, and those for the neutral targets are represented by square markers connected by solid lines. In Figs. (**b**)**–**(**d**), the black columns indicate the “target emotion effects,” derived from the differences on (**b**) efficacy, (**c**) latency, or (**d**) imprecision values between the negative-target condition and the neutral-target condition, with a positive value indicating an effect favoring the negative-target condition. The error bars indicate 95% CIs of the target emotion effects

The SPE distributions for the one-target/neutral, the one-target/negative, the two-target/neutral, and the two-target/ negative conditions are shown in Fig. 6a, with the error bars indicating the 95% CIs across the 25 participants. Figure 6b–d show the mean efficacy, latency, and imprecision values in each of the four aforementioned conditions. The black columns in Fig. 6b–d indicate the “target emotion effects,” derived from the differences between the neutral-target condition and the negative-target condition, where a positive value indicates an effect favoring the negative-target condition. The statistical analyses are shown below.

- *Efficacy*. There was a substantial effect of target number (*F*(1, 24) = 108.5, *p* < .001, *η*_*partial*_^*2*^ = .82) manifested by a higher efficacy value in the one-target condition (*M* = .78) than the two-target condition (*M* = .49). The target emotion also yielded a significant effect (*F*(1, 24) = 13.57, *p* = .001, *η*_*partial*_^*2*^ = .36), caused by higher efficacy values for the negatively charged target characters (*M* = .65) than neutral characters (*M* = .62). The effect of target side, and any significant interaction among target side, target emotion, and target number did not reach statistical significance (all *ps*>.05, all *η*_*partial*_^*2’*^*s* < .15). The 95% CIs of the left-side efficacy advantages of the one-target condition and the two-target condition were, respectively, [-0.02, 0.02] and [-0.07, 0.11], where a positive value indicates an effect favoring the left side.
- *Latency*. The main effect of target number, target emotion, target side, and any interaction among the three factors did not reach statistical significance (all *ps*>.05, all *η*_*partial*_^*2’*^*s* < .15). The 95% CIs of the left-side latency advantages of the one-target condition and the two-target condition were, respectively, [-18 ms, 8 ms] and [-30 ms, 43 ms], where a positive value represents an effect favoring the left side.
- *Imprecision*. There was a significant effect of target number (*F*(1, 24) =11.52, *p* = .002, *η*_*partial*_^*2*^ = .32), caused by lower selection imprecision (more precise) in the one-target condition (*M* = 68 ms) than the two-target condition (*M* = 91 ms). The effects of target emotion, target side, and any interaction among target side, target emotion, and target number did not reach statistical significance (all *ps*>.05, all *η*_*partial*_^*2’*^*s* < .15). The 95% CIs of the left-side imprecision advantage of the one-target condition and the two-target condition were, respectively, [-11 ms, 24 ms] and [-22 ms, 23 ms], where a positive value represents an effect favoring the left side.

#### Two-target condition only

The efficacy, latency, and imprecision values were subjected to a repeated-measures ANOVA, with three within-subject factors of target emotion (neutral or negative), target side (left or right), and neighbor emotion (neutral or negative).

**Fig. 7:**
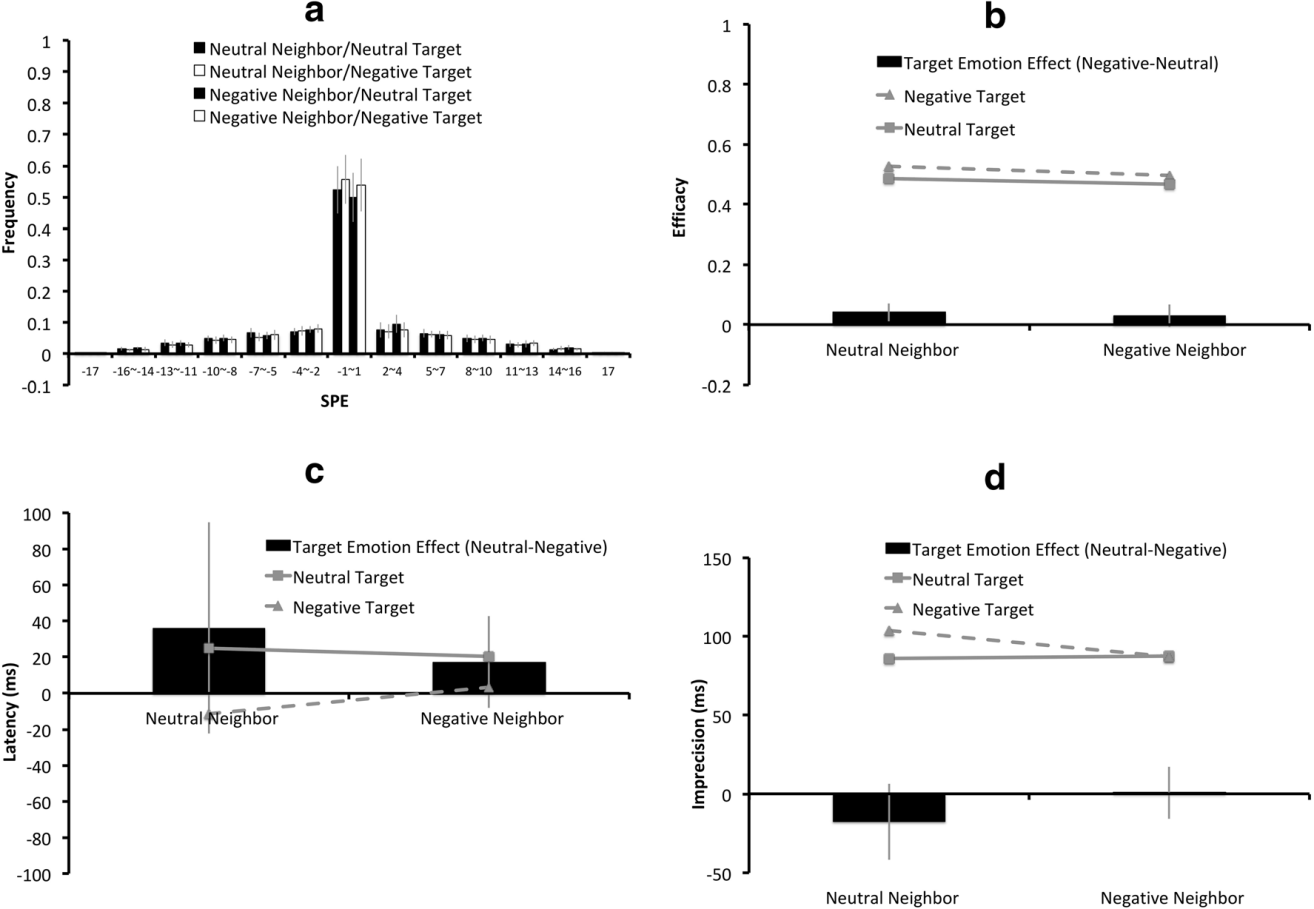
Results of the two-target condition only in Experiment 3: (**a**) The serial position error (SPE) distributions for the neutral-neighbor/neutral-target, the neutral-neighbor/negative-target, the negative-neighbor/neutral-target, and the negative-neighbor/negative–target conditions are plotted from left to right for each SPE bin. The (**b**) mean efficacy, (**c**) latency, and (**d**) imprecision values for the negatively charged targets are represented by triangular markers connected by dashed lines, and those for the neutral targets are represented by square markers connected by solid lines. In Figs. (**b**) **–**(**d**), the black columns indicate the “target emotion effects,” derived from the differences on the (**b**) efficacy, (**c**) latency, or (**d**) imprecision values between the negative-target condition and the neutral-target condition, with a positive value indicating an effect favoring the negative-target condition. The error bars indicate 95% CIs of the target emotion effects

The SPEs plotted for each neighbor-target emotion combination (neutral-neutral, neutral-negative, negative-neutral, and negative-negative) are shown in Fig. 7a, with the error bars indicating the 95% CIs across the 25 participants. Figure 7b–d illustrate the means for each neighbor-target emotion combination on efficacy, latency and imprecision. The black columns in Fig. 7b–d represent the “target emotion effects,” derived from the differences between the efficacy (Fig. 7b), latency (Fig. 7c), or imprecision (Fig. 7d) values in the neutral-target condition and the negative-target condition.

- *Efficacy.* There was a significant effect of target emotion (*F*(1, 24) = 8.6, *p* = .007, *η*_*partial*_^*2*^ = .26), caused by higher efficacy values for negatively charged targets (*M* = .51) than for neutral targets (*M* = .48). There was also a significant effect of neighbor emotion (*F*(1, 24) = 5.99, *p* = .02, *η*_*partial*_^*2*^ = .20), caused by higher efficacy values for characters with neutral neighbors (*M* =.51) than characters with negatively charged neighbors (*M* =.48). There was no significant main effect of target side, nor any interaction among target emotion, neighbor emotion, and target side (all *ps*>.05, all *η*_*partial*_^*2’*^*s* < .15).
- *Latency and imprecision*. There was no significance main effect of target emotion, neighbor emotion, target side, nor any interaction among the three factors (all *ps*>.05, all *η*_*partial*_^*2’*^*s* < .15) on latency and imprecision.

#### Rating results

Similar to Experiment 2, a between-subject *t-*test (with each character treated as a “subject”) on the emotional positivity rating values derived from the 25 participants showed that the 120 negatively charged characters were indeed perceived to be more negative (*M* = 2.74) than the 120 neutral characters (*M* = 4.27) (*t(238)* = 26.89, *p* < .0001, *Cohen’s d* = 3.47).

#### Concreteness

Using linguistic materials as emotional stimuli might potentially involve unwanted confounding variables. For example, one might argue that the effects of emotion observed in the current study might have confounded with character concreteness. We did not control this factor in advance, so could only conduct a *post hoc* analysis to examine the effect of concreteness. After the completion of Experiments 2 and 3, we designed an online questionnaire aiming to test people’s perception of concreteness of each character used in this study. The questions were framed in a way similar to the questions used in the study of Yao, Wu, Zhang, and Wang (2017), where participants had to judge how likely a particular word could provoke a thought about a concrete object or a scene, on a 7-point Likert scale. One hundred native Chinese speakers filled out this questionnaire online. Statistically, negatively charged characters were indeed perceived to be more concrete than neutral characters (*t (238)* = 2.59, *p* =.01), but the effect size was fairly small (negative: *M =* 4.49; neutral: *M* = 4.18; *Cohen’s d* = 0.33), which was substantially smaller than the effect sizes of emotional positivity rating values in Experiment 2 (*Cohen’s d* = 3.46) and Experiment 3 (*Cohen’s d* = 3.47).

If the emotional effect observed in this study had been confounded with character concreteness, there should have been a correlation between the participant’s task performance for each character, which could be indexed by the absolute SPE (unsigned serial position error), and the index of concreteness for each target. Thus, we did a correlational analysis between character concreteness rating values and absolute SPEs across all targets for each participant, and found that the average correlational coefficients did not significantly differ from zero in Experiments 2 and 3 (*ts <* 2.1, *ps* >.05). However, the average correlation coefficients between the character emotional positivity rating values and absolute SPEs were significantly larger than zero in Experiments 2 and 3 (*ts>* 2.8, *ps* <.01), showing that the more negative the target character was, the smaller absolute SPE value the target character induced. Although negatively charged characters were perceived to be slightly more concrete than neutral targets, the degree of concreteness of the character did not significantly predict its induced task performance, whereas the degree of emotional positivity did.

### Discussion

In this experiment, target presentation time was increased so more time was available for consolation. Indeed, the efficacy for negatively charged targets was significantly higher than that for neutral targets, showing a negative superiority effect. Interestingly, this negative superiority effect for target emotion was accompanied by a negative inferiority effect for neighbor emotion: Having a negative neighbor decreased the processing efficacy. This implied that consolidation is subject to limited resources. Resources are more likely to be deployed to negatively charged characters, causing a disruptive effect for their neighbors.

## General discussion

### Summary of results

In Experiment 1, we replicated Goodbourn and Holcombe’s (2015) study. When viewing one RSVP stream composed of English letters, there was no significant effect of target number or target side on selection latency or imprecision; when viewing two RSVP streams, an efficacy benefit favoring the left side was manifested. In Experiment 2, where Chinese characters were used, latency effects induced by target emotion and neighbor emotion were observed. Either being a negatively charged target or having a negatively charged neighbor led to shortened selection latency. It is possible that attending to negatively charged stimuli triggered some subcortical neural circuits (Pop-Jordanov & Pop-Jordanova, 2009) that accelerated the attentional process. However, there was no significant effect on efficacy in the two-target conditions. In Experiment 3, the stimulus duration increased. The latency effect became insignificant, but an efficacy benefit favoring negatively charged targets emerged. Interestingly, the efficacy benefit for negatively charged targets was accompanied by a cost for having negatively charged neighbors.

There was an unexpected finding that the imprecision value for the one-target condition was lower than that for the two-target condition in Experiment 3. Given that emotion could modulate selection latency, as shown in Experiment 2, the trial-to-trial variability on stimulus emotion should also affect selection imprecision. For the one-target condition, the target could be neutral or negative; for the two-target condition, the target pair could be neutral versus neutral, neutral versus negative, negative versus neutral, or negative versus negative. The higher trial-to-trial variability on stimulus emotion might be the cause for the less precise selection process in the two-target condition than the one-target condition.

### Comparison between Experiment 2 and Experiment 3

Both target emotion and the neighbor emotion yielded significant effects on latency in Experiment 2, but neither did in Experiment 3. One possibility could be that the short target presentation time in Experiment 2 provided a scale of high temporal resolution for different stimulus emotions (negative and neutral) to exert differential effects on selection latency. In Experiment 3, there was still a trend that the latency value decreased as long as the target or the neighbor was negative (Fig. 7c), but it did not reach statistical significance. Possibly, the selection mechanism induced by neutral items was still slower than that induced by negative items, but each item was presented too long so even a lagged selection mechanism still frequently selected the same item as the leading selection mechanism. Thus, the effect size of latency would be underestimated.

When there were two targets, the higher efficacy values induced by negatively charged targets, together with the lower efficacy values induced by negatively charged neighbors, were only statistically significant in Experiment 3 (Fig. 7b). In Experiment 2, one could still find the same trend (Fig. 5b), which failed to reach statistical significance. Possibly, the long target presentation time in Experiment 3 enabled different emotions to induce differential effects at the consolidation stage, and thus the emotional effect on efficacy was more likely to be observed in Experiment 3. In the case of Experiment 2, the short presentation time allowed too few items to reach the consolidation stage for the manifestation of the emotional effect.

In regard to imprecision, the one-target condition yielded a lower imprecision value (more precise) than the two-target condition in Experiment 3, but this effect was not significant in Experiment 2. The larger data range of Experiment 3 might have provided a more sensitive measurement for any imprecision effect to emerge. Possible SPEs in the present study ranged from −17 to 17, which corresponded to −1,600 ms to 1,600 ms in Experiment 2 and −2,600 to 2,600 ms in Experiment 3. The absence of a significant target number effect on imprecision in Experiment 2 was possibly due to the narrower data range that might have truncated some imprecision values, thus reducing the effect size.

### Insignificant target side effect for Chinese characters

One thing to note is that the significant left-side advantage for the English letters became statistically insignificant for Chinese characters. In fact, the target side effect was highly related to the implied reading direction of the stimuli. Even for the English letters, the significant left-side advantage was eliminated when the letters were mirrored or rotated to face to the left (Holcombe, Nguyen, & Goodbourn, 2017). Moreover, when English-Arabic bilinguals were tested with English letters, they showed a left-side advantage as English readers, but when tested with Arabic letters, a trend towards a right-side advantage was observed (Ransley, Goodbourn, Nguyen, Moustafa, & Holcombe, 2018). Chinese characters can be read both from left to right, and from right to left, depending on the context. When there is little contextual cue, as in the experiments of the present study, participants can read in both directions, leading to the absence of a significant target side effect.

### How does emotion interact with attention?

The emotional effect on perception has been shown to be facilitative in some studies (Öhman et al., 2001; Phelps et al., 2006; Pourtois et al., 2004; Pourtois et al., 2005; Van Damme et al., 2008) and inhibitory in others (Kennedy et al., 2014; Most et al., 2006; Most et al., 2005; Most & Jungé, 2008; Wang et al., 2012). Here, we offer an explanation for the contradictory effects of emotion:

#### Emotion accelerates attentional selection

When observers are required to select a set of stimuli in the visual display, the overall selection speed can be accelerated if the potential targets include emotional stimuli, regardless of the stimulus location. This is why the selection latency reduced as long as the target stream, or the neighbor stream contained negatively charged characters. It is possible that the presence of a negatively charged character elevated the participant’s arousal state, which then triggered thalamocortical interactions involving reticular-limbic and thalamic excitatory control systems (Pop-Jordanov & Pop-Jordanova, 2009). This subcortical interaction is not location-specific, so the latency effect is not restricted to the target location only. The “acceleration” effect of emotion might manifest itself in different forms. In tasks that require rapid identification, an accelerated process could lead to better identification performance, as in the case of Phelps et al. (2006), where a centrally presented emotional face picture facilitated the identification performance of the peripherally presented targets.

#### Emotional stimuli are prioritized for consolidation

After potential target representations have been selected, they must undergo the consolidation process for conscious report (Chun & Potter, 1995; Goodbourn & Holcombe, 2015; Kanwisher, 1987), and emotional stimuli are prioritized for consolidation. Because attentional resources for consolidation are limited, a negative superiority effect for targets will be compensated by a negative inferiority effect for non-targets. For target emotion, negatively charged items can be consolidated better, possibly mediated by direct projections from the amygdala to visual cortical areas (Vuilleumier, Richardson, Armony, Driver, & Dolan, 2004). For neighbor emotion, having a negatively charged neighbor impairs the efficacy because the limited attentional resources (Forster & Lavie, 2008; Lavie, 1995) are depleted by the negatively charged neighbor. This resource depletion effect could account for previous studies that showed an EIB effect: The presentation of an emotional distracter depleted attentional resources for the consolidation of the target.

Bocanegra and Zeelenberg's (2009) study demonstrated that cue-target stimulus-onset asynchrony (SOA) was a critical factor. The emotional effect was inhibitory with short cue-target SOAs (50 ms and 500 ms), but facilitative with a long cue-target SOA (1000 ms). In the present study, however, emotional characters induced an overall facilitative effect on selection latency with a short stimulus presentation time, and location-dependent facilitative and inhibitory effects on efficacy with a long presentation time. Note that the cue and the target were presented separately in Bocanegra and Zeelenberg's (2009) study, but simultaneously in the present study. Experiment 2 of the present study might have probed a mechanism equivalent to a similar one probed by 0~59 ms cue-target SOAs, and Experiment 3 might have probed a mechanism equivalent to a similar one probed by 0- to 118-ms cue-target SOAs. According to Bocanegra and Zeelenberg's (2009) study, inhibitory effects should have been found in both Experiments 2 and 3 because they both involved SOAs shorter than 500 ms. Indeed, there was an inhibitory effect from the emotional neighbor to the target in Experiment 3 in the present study. Experiment 2 did not show a significant neighbor emotion effect, possibly due to the high task difficulty in this study that only enabled too few items to be successfully consolidated. In terms of the facilitative selection latency effect, it could be long lasting, starting from 0~59 ms SOAs, as in the case of Experiment 2 of the present study, until 1,000 ms, as in the case of Bocanegra and Zeelenberg's (2009) study. However, this facilitative effect could be masked by the emotion-induced inhibition in the consolidation stage in the task used in Bocanegra and Zeelenberg's (2009) study, leading to an inhibitory net effect with SOAs of 50 ms and 500 ms.

### Conclusions

The present study, based on the experimental paradigm developed by Goodbourn and Holcombe (2015), has offered a comprehensive model to account for the dual role of emotion on perception. Firstly, emotion accelerates the selection process, possibly through subcortical interactions, and this effect is facilitative and global. This was evidenced by the shortened latency value for being a negatively charged target or having a negatively charged neighbor in Experiment 2. Secondly, emotional stimuli are prioritized for consolidation, which requires attentional resources. For simultaneously presented stimuli, the emotionally charged ones attract attention to their locations, depleting the resources for other locations. This was evidenced by higher efficacy for negatively charged targets with lower efficacy for having negatively charged neighbors in Experiment 3, where the target presentation time was relatively long for emotional effects at late stages of information processing to manifest.

## Appendix

**Table 1.** Characters used in Experiments 2 and 3

Characters	Meanings					Characters	Meanings				
Negative	Negative					Neutral	Neutral				
婊 謊 賠 殘 毒	whore	lie	indemnify	disabled	poison	咨 弓 頒 涯 章	consult	bow	confer	edge	chapter
孬 墮 笨 遜 惡	coward	degenerate	stupid	inferior	evil	槳 焉 曆 榜 課	paddle	how	calendar	placard	lesson
訃 詐 屁 幹 危	obituary	defraud	fart	fuck	danger	澗 筷 埔 軸 塊	brook	chopstick	plain	axis	lump
娼 貶 悔 憂 操	prostitute	belittle	regret	grief	fuck	縝 酋 蓬 宿 州	detailed	chief of tribe	fluffy	lodge	administrative division
迂 凶 茫 奪 怪	tortuous	fierce	unclear	rob	strange	釦 吾 湘 譜 築	button	I	Hunan province	musical score	build
閹 屍 欺 盜 殺	castrate	corpse	bully	thief	kill	榫 亭 裔 輔 伴	tenon	pavilion	ethnicity	support	partner
剁 棺 羞 魂 癌	chop	coffin	shame	phantom	cancer	扈 鈞 毅 泉 析	escort	a unit of measure	perseverance	fountain	analyze
殯 匪 悶 滾 退	funeral	robber	stuffy	roll	withdraw	蜀 矽 瓷 悉 幅	Sichuan province	silicon	porcelain	comprehend	(quantifier for paintings)
扒 滯 罵 鬥 急	snatch	stagnant	scold	fight	urgent	扉 沐 棟 衡 範	door panel	bathe	(quantifier for buildings)	balance	model
屎 妒 罰 貧 苦	feces	jealous	punish	poverty	bitter	棗 菩 銜 址 窗	date	Bodhisattva	bite	address	window
姦 畜 呆 鬼 痛	rape	beast	foolish	ghost	pain	棠 羽 撰 貿 域	(plant name)	feather	compose	trade	field
奸 妓 醜 逃 背	mean	prostitute	ugly	escape	unlucky	磐 划 浩 閱 票	large	rock	row	vast	read ticket
斃 泣 劣 弱 缺	kill	sob	inferior	weak	lack	絮 萃 浦 堡 策	cotton fiber	extract	shore	fort	plan
忿 賊 愁 混 輸	rage	thief	worry	slack	lose	煦 桶 逸 遍 校	warm	bucket	relaxed	spread	school
悵 冤 恨 暴 負	disappointed	injustice	hatred	violence	negative	磊 辰 廂 序 育	rocks	morning	cubicle	order	educate
糞 淫 凸 悲 害	feces	promiscuous	protrude	sad	harm	筵 軒 罩 箱 輪	feast	small room	cover	box	wheel
褻 奴 哭狂差	blasphemy	slave	cry	mad	bad	茉 矩 樑 鼓 居	jasmine	ruler	beam	drum	reside
惰 拙 慘 禁 黑	lazy	clumsy	catastrophic	forbidden	black	苔 株 函 授 普	moss	(quantifier for plants)	envelop	teach	general
惘 頑 忌 疾 死	confused	stubborn	jealous	sick	death	亥 郊 埃 宇 局	9 pm~11 pm	suburbs	dirt	universe	circumstance
咒 啞 喪 棄 換	curse	mute	funeral	abandon	change	崙 昨 杯 偶 房	(mountain name)	yesterday	cup	coincidently	house
剋 葬 偷 誤 傷	overcome	bury	steal	mistake	injury	茁 澄 邦 席 似	flourish	clear	state	seat	resemble
噓 糟 墓 亂 絕	hiss	crap	tomb	chaotic	terminate	臼 寓 晨 琴 率	mortar	apartment	morning	musical instrument	lead
賤 傻 怨 亡 失	degraded	silly	complain	perish	miss	哉 扶 涉 詞 曲	(exclamatory article)	hold	involve	words	melody
瞎 憤 廢 敗 老	blind	resent	useless	failure	old	咸 晴 桿 寧 話	all	sunny	pole	serenity	speech
Mean Character Frequency						Mean Character Frequency					
92						91					
Mean Stroke Count						Mean Stroke Count					
11						11					

1 There was no significant difference on character frequency (t(238) = 0.07, p = .95) and stroke count (t(238) = 0.12, p = .90) between the negatively charged characters and neutral characters

2 The character frequency is based on the database of National Language Committee, Ministry of Education of Taiwan (1998), and is expressed as number of occurrences per million

## Footnotes

1 In Goodbourn and Holcombe’s (2015) paper, they used “precision” instead of “imprecision” to refer to the standard deviation of the Gaussian distribution. However, the term “precision” could be misleading because a higher standard deviation in the Gaussian distribution actually indicated a less precise selection process. Therefore, “imprecision” is used in this paper to match the effect direction of the standard deviation.

2 In this analysis, we chose the “t-test” family, which compared the difference between two dependent means. The α value was set to .05, dz was set to 1.81, the number of tails was set to 2, and the power value was set to .9.

3 This analysis was based on the data from Experiment 1 in Most and Wang’s (2011) study. The mean accuracy of the perceptual judgment task was 57.37% and 70.31%, respectively, in the negative condition and the neutral condition, and the corresponding t value was 3.57. Based on the t-value provided, the dz value could be derived, together with the α value set to .05, the number of tails set to 2, and the power value set to .9, the total sample size should be 16, according to the power analysis with G*Power 3.
